# Supplementary material for: Evaluating Machine Learning-Based Classification of Human Locomotor Activities for Exoskeleton Control Using Inertial Measurement Unit and Pressure Insole Data
Source: Sensors (Basel). 2025 Aug 29;25(17):5365. doi: 10.3390/s25175365 (PMC12431005; doi:10.3390/s25175365)
Supplement: Supplementary file 1 [file sensors-25-05365-s001.zip › sensors-3802860-supplementary.pdf]

Supplementary Materials

Table S1. Rank ordered feature importances for statistic, IMU signal, and IMU or insole sensor location parameters in the RF and LGBM models for speed and incline regression. RF scores are the normalised (0-1) decrease in impurity, representing the relative proportion of total importance. LGBM scores are the raw count of times a feature is used for splits across all trees.

	Rank	RF		LGBM	
		Speed	Incline	Speed	Incline
Statistics	1	Min (0.51)	Max (0.53)	Min (350)	Min (390)
	2	Max (0.49)	Min (0.46)	Max (350)	Max (310)
Signal	1	Gyro (0.78)	G Angle (0.32)	Gyro (277)	Gyro (252)
	2	Accel (0.13)	Gyro (0.28)	Mag (193)	G Angle (150)
	3	G Angle (0.05)	Mag (0.17)	G Angle (99)	Mag (135)
	4	Mag (0.04)	Accel (0.13)	Accel (74)	Accel (109)
Sensor	1	R. Shank (0.2)	R. Thigh (0.24)	L. Thigh (124)	R. Thigh (174)
	2	R. Thigh (0.19)	Pelvis (0.2)	R. Thigh (110)	Pelvis (116)
	3	L. Thigh (0.19)	L. Thigh (0.13)	L. Shank (105)	L. Thigh (111)
	4	L. Shank (0.15)	R. Shank (0.11)	Pelvis (95)	L. Shank (83)
	5	R. Foot (0.1)	L. Shank (0.08)	L. Foot (76)	R. Foot (64)
	6	L. Foot (0.1)	L. Foot (0.07)	R. Foot (68)	R. Shank (59)
	7	Pelvis (0.05)	R. Foot (0.07)	R. Shank (65)	L. Foot (39)
	8	L. Insole (0.00)	R. Insole (0.04)	L. Insole (32)	L. Insole (30)
	9	R. Insole (0.00)	L. Insole (0.04)	R. Insole (25)	R. Insole (24)

*Min - minimum; Max – maximum; Gyro – gyroscope; Accel – accelerometer; G Angle – global angle; Mag - magnetometer

Table S2. Parameter values resulting from the random search cross-validation tuning, subsequently used in the experiments. These values, along with the trained models themselves, are also available in the code repository.

Model Type	Parameter	Value
LR, RF, LGBM	Sliding Window Size	800
	Train-validation Split	0.8
RF	max_depth	20
	max_features	Sqrt
	min_samples_leaf	8
	min_samples_split	8
	n_estimators	28
	bootstrap	true
LGBM	bagging_fraction	0.6
	bagging_freq	5
	extra_trees	True
	feature_fraction	0.6
	lambda_l1	0.0
	lambda_l2	0.1
	max_bin	255
	max_depth	3
	min_data_in_leaf	50
	min_gain_to_split	0.1
	min_sum_hessian_in_leaf	0.1
	num_leaves	63
	path_smooth	0.3
